# A Core MRB1 Complex Component Is Indispensable for RNA Editing in Insect and Human Infective Stages of *Trypanosoma brucei*

**DOI:** 10.1371/journal.pone.0078015

**Published:** 2013-10-18

**Authors:** Michelle L. Ammerman, Danielle L. Tomasello, Drahomíra Faktorová, Lucie Kafková, Hassan Hashimi, Julius Lukeš, Laurie K. Read

**Affiliations:** 1 Department of Microbiology and Immunology, University at Buffalo School of Medicine, Buffalo, New York, United States of America; 2 Institute of Parasitology, Biology Center, Czech Academy of Sciences and Faculty of Science, University of South Bohemia, České Budějovice (Budweis), Czech Republic; University of Texas Medical School at Houston, United States of America

## Abstract

Uridine insertion/deletion RNA editing is a unique and vital process in kinetoplastids, required for creation of translatable open reading frames in most mitochondrially-encoded RNAs. Emerging as a key player in this process is the mitochondrial RNA binding 1 (MRB1) complex. MRB1 comprises an RNA-independent core complex of at least six proteins, including the GAP1/2 guide RNA (gRNA) binding proteins. The core interacts in an RNA-enhanced or -dependent manner with imprecisely defined TbRGG2 subcomplexes, Armadillo protein MRB10130, and additional factors that comprise the dynamic MRB1 complex. Towards understanding MRB1 complex function in RNA editing, we present here functional characterization of the pentein domain-containing MRB1 core protein, MRB11870. Inducible RNAi studies demonstrate that MRB11870 is essential for proliferation of both insect vector and human infective stage *T. brucei*. MRB11870 ablation causes a massive defect in RNA editing, affecting both pan-edited and minimally edited mRNAs, but does not substantially affect mitochondrial RNA stability or processing of precursor transcripts. The editing defect in MRB1-depleted cells occurs at the initiation stage of editing, as pre-edited mRNAs accumulate. However, the gRNAs that direct editing remain abundant in the knockdown cells. To examine the contribution of MRB11870 to MRB1 macromolecular interactions, we tagged core complexes and analyzed their composition and associated proteins in the presence and absence of MRB11870. These studies demonstrated that MRB11870 is essential for association of GAP1/2 with the core, as well as for interaction of the core with other proteins and subcomplexes. Together, these data support a model in which the MRB1 core mediates functional interaction of gRNAs with the editing machinery, having GAP1/2 as its gRNA binding constituents. MRB11870 is a critical component of the core, essential for its structure and function.

## Introduction

The mitochondrial genome of the kinetoplastid flagellate *Trypanosoma brucei* is comprised of dozens of maxicircles and thousands of minicircles that are concatenated into a dense network [1]. Maxicircles contain 18 protein-encoding genes, the majority of which represent subunits of the respiratory chain, as well as two rRNAs. Two-thirds of maxicircle-encoded mRNAs require dramatic remodeling by specific uridine (U) insertion/deletion RNA editing for the creation of translatable open reading frames. This process is essential for both insect procyclic form (PF) and mammalian bloodstream form (BF) *T. brucei* [2,3]. A few mRNAs are edited only within discrete regions, and are referred to as minimally-edited. However, in the majority of mRNAs, termed pan-edited, the degree of U insertion is so extreme that it doubles the size of the primary transcript. *Trans*-acting guide RNAs (gRNAs), primarily encoded within the minicircle components of the mitochondrial DNA network, specify the precise insertion and deletion of Us into mRNAs through gRNA/mRNA base-pairing interactions. The enzymatic reactions of the RNA editing process (endonuclease, terminal uridylyl transferase, U-specific 3’ exoribonuclease and RNA ligase activities) are catalyzed by multiprotein complexes termed editosomes or RNA editing core complexes (RECCs) [3,4]. 

 In addition to the editosome, several non-editosome proteins facilitate the editing of one or more mRNAs *in vivo* [5-11]. Most importantly, a large, dynamic macromolecular complex termed the MRB1 (mitochondrial RNA binding 1) complex, otherwise known as GRBC (gRNA binding complex), has recently emerged as essential for mitochondrial RNA editing [10]. Three groups originally isolated MRB1 through immunoaffinity purification of the GAP1 or GAP2 proteins (for gRNA associated proteins; a.k.a. GRBC1 and 2) [9,12,13]. Purifications of GAP1/2 associated proteins performed in different laboratories resulted in overlapping, but substantially different, sets of proteins. We recently elucidated the architecture of the MRB1 complex and defined its component subcomplexes through a combination of directed yeast two-hybrid screen and *in vivo* pulldowns with numerous endogenously tagged proteins [14]. We found that MRB1 contains a core associating in an RNA-independent manner, composed of GAP1/2 and the MRB11870, MRB3010, MRB5390 and MRB8620 proteins (nomenclature based on TriTrypDB numbers; http://tritrypdb.org/tritrypdb/). GAP1/2, which function as a heterotetramer that binds and stabilizes gRNA [15], may interact with the rest of the core in a dynamic fashion and thereby serve to deliver gRNA to the core. These two proteins are essential for RNA editing, presumably due to their gRNA stabilizing function [13,16]. Repression of the core protein MRB3010 also causes a broad disruption of RNA editing; however, this takes place in the face of normal gRNA levels [17]. Moreover, in the MRB3010 knockdown cells, RNA editing is impaired at an early step in the process, as shown by the accumulation of pre-edited mRNAs, but not partially edited mRNAs. Repression of core protein MRB5390 also disrupts editing, although to a lesser extent as compared to the MRB3010 knockdown [18]. Collectively, the current data are consistent with a model in which the MRB1 core is critical for the functional association of gRNA with the editosome, and GAP1/2 are the gRNA-binding components of the core.

 A second important component of the MRB1 complex is TbRGG2, an RNA binding protein that forms mutually exclusive subcomplexes with two paralogous proteins MRB8170 and MRB4160, and also interacts with other proteins including MRB8180 [19-22]. Repression of TbRGG2 dramatically affects editing of pan-edited mRNAs, but does not affect minimally-edited mRNAs [18,19]. TbRGG2 silencing modestly impacts editing initiation, but has a more significant effect on the 3’ to 5’ progression of editing along pan-edited mRNAs, resulting in the accumulation of partially edited RNAs [23]. Recombinant TbRGG2 preferentially binds pre-edited mRNAs compared to edited mRNAs or gRNAs and catalyzes gRNA/mRNA annealing [20]. *In vivo* complementation studies implicate the RNA annealing activity of the protein as being essential for RNA editing [20]. The TbRGG2-associated proteins, MRB8170 and MRB4160, are paralogues with redundant functions in editing that also impact pan-edited mRNAs to a greater degree than minimally edited mRNAs [21]. These proteins both bind RNA, and like TbRGG2, display a marked preference for mRNA over gRNA [20,21]. TbRGG2/MRB8170/MRB4160 subcomplexes may be multifunctional, as they have also been reported in association with the gRNA processing endonuclease, mRPN1 [22]. Together, results to date implicate the TbRGG2 subcomplex(es) in productive gRNA/mRNA interaction, especially during editing progression. 

 An additional protein that is present in the majority of MRB1 purifications is MRB10130, a protein almost entirely composed of Armadillo repeats. In yeast two-hybrid screens, MRB10130 exhibits weak interactions with a large number of MRB1 components and a strong direct interaction with TbRGG2 [14]. As Armadillo proteins often act as organizers of multiprotein complexes, our data suggest that MRB10130 may play a similar role, organizing interactions between the MRB1 core, TbRGG2 subcomplexes, and additional subcomplexes and proteins that comprise the dynamic MRB1 complex. All components of the MRB1 complex that have been tested thus far are essential for growth of PF *T. brucei* (the paralogous MRB8170/4160 are redundant such that only the double knockdown impairs growth) [10,13,16-19,21]. Those that have been examined in BF are essential for growth in this life cycle stage as well [16,17,19].

In addition to the essential functions of the MRB1 complex in RNA editing, numerous lines of evidence link MRB1 to other aspects of mitochondrial gene regulation such as RNA maturation, stability and translation [10]. For example, *in vivo* pulldown experiments demonstrate an interaction between enzymes involved in mRNA 3’ tail addition and the MRB1 complex, namely KPAP1, a mitochondrial poly(A) polymerase, and KPAF1, a PPR-motif bearing RNA-binding protein [13,24,25]. Accordingly, yeast two-hybrid studies identified direct interactions between KPAF1 and both the MRB1 core and TbRGG2 subcomplex(es) [14]. The MRB1 complex also occasionally co-purifies with MERS1, a NUDIX hydrolase domain-containing protein thought to be involved in stabilizing edited mRNAs [9,13]. Finally, substoichiometric levels of several ribosomal proteins are often identified in association with MRB1, and a more detailed study demonstrated the association of the GAP1/2 proteins with the large ribosomal subunit [26]. Thus, the MRB1 complex connects RNA editing to other aspects of mitochondrial gene expression in a manner whose temporal and spatial details are yet to be uncovered.

In this study, we present a functional characterization of an MRB1 complex protein, MRB11870. Previous *in vivo* pulldown experiments clearly demonstrated that MRB11870 is a component of the MRB1 complex core as it engages in RNA-independent interactions with numerous core components [14]. Yeast two-hybrid studies identified a strong direct interaction of this protein only with MRB3010, suggesting some of these interactions are likely indirect. MRB11870 also engages in weak two-hybrid interactions with the core protein GAP1 and the Armadillo repeat protein MRB10130 [14]. Importantly, the function of MRB11870 cannot be implied simply from its residence in the MRB1 core, as RNAi studies mentioned above reveal different phenotypes upon knockdown of different core components. Repression of GAP1/2 leads to destabilization of the entire gRNA population and a resultant halt in editing [13,16]. In contrast, knockdown of MRB3010 does not affect gRNA levels, but severely impacts editing of the majority of mRNAs [17], while MRB5390 repression has a more modest effect on RNA editing [18]. Here, we show that MRB11870 is essential for growth of both PF and BF *T. brucei*. Quantitative (q) RT-PCR analyses demonstrate that MRB11870 repression leads to a very strong editing defect that impacts both pan-edited and minimally-edited mRNAs. Editing in MRB11870-ablated cells appears to be halted at an early step in the process, although gRNAs remain abundant, similar to the phenotype observed upon repression of the core protein, MRB3010. Analysis of protein-protein interactions in MRB11870-depleted cells demonstrates that this protein is critical for association of GAP1/2, MRB10130, and TbRGG2 subcomplexes with the core. Thus, MRB11870 is a key protein involved in numerous structural interactions of the core as well as in initial stages of editing in both human and insect stage *T. brucei*.

## Results

### MRB11870 is a pentein protein highly conserved among Kinetoplastida

MRB11870 is a kinetoplastid-specific protein of 310 amino acids with a predicted molecular mass of 34.7 kDa and pI of 6.7. It is highly conserved across the kinetoplastid flagellates, exhibiting 92% identity/95% similarity to its *Trypanosoma cruzi* homologue and 64% identity/78% similarity to its homologue in *Leishmania major*. Divergence between the *T. brucei* and *L. major* proteins stems in large part from an 18 amino acid-long insertion in the latter. The bulk of the protein, with the exception of the N terminal 12 and C terminal 27 residues, comprises a pentein domain (Superfamily SSF55909, E value 5.1x10^-15^). Pentein superfamily proteins are defined by their similar beta/alpha propeller structural folds [27]. The family contains both catalytic proteins, including many proteins that modify guanidines, as well as non-catalytic proteins such as eukaryotic initiation factor 6 (eIF6), which plays key roles in both 60S ribosomal subunit assembly and preventing premature association of 60S and 40S subunits during translation [27,28]. Since the pentein fold arises from disparate primary protein sequences, and this domain plays several catalytic and structural roles in the cell, it is difficult to assign a function to MRB11870 based on this information alone [27]. Furthermore, BLAST searches performed with MRB11870 do not identify any convincing homologues outside the Kinetoplastida.

### MRB11870 is essential for growth in PF and BF trypanosomes

**Figure 1:**
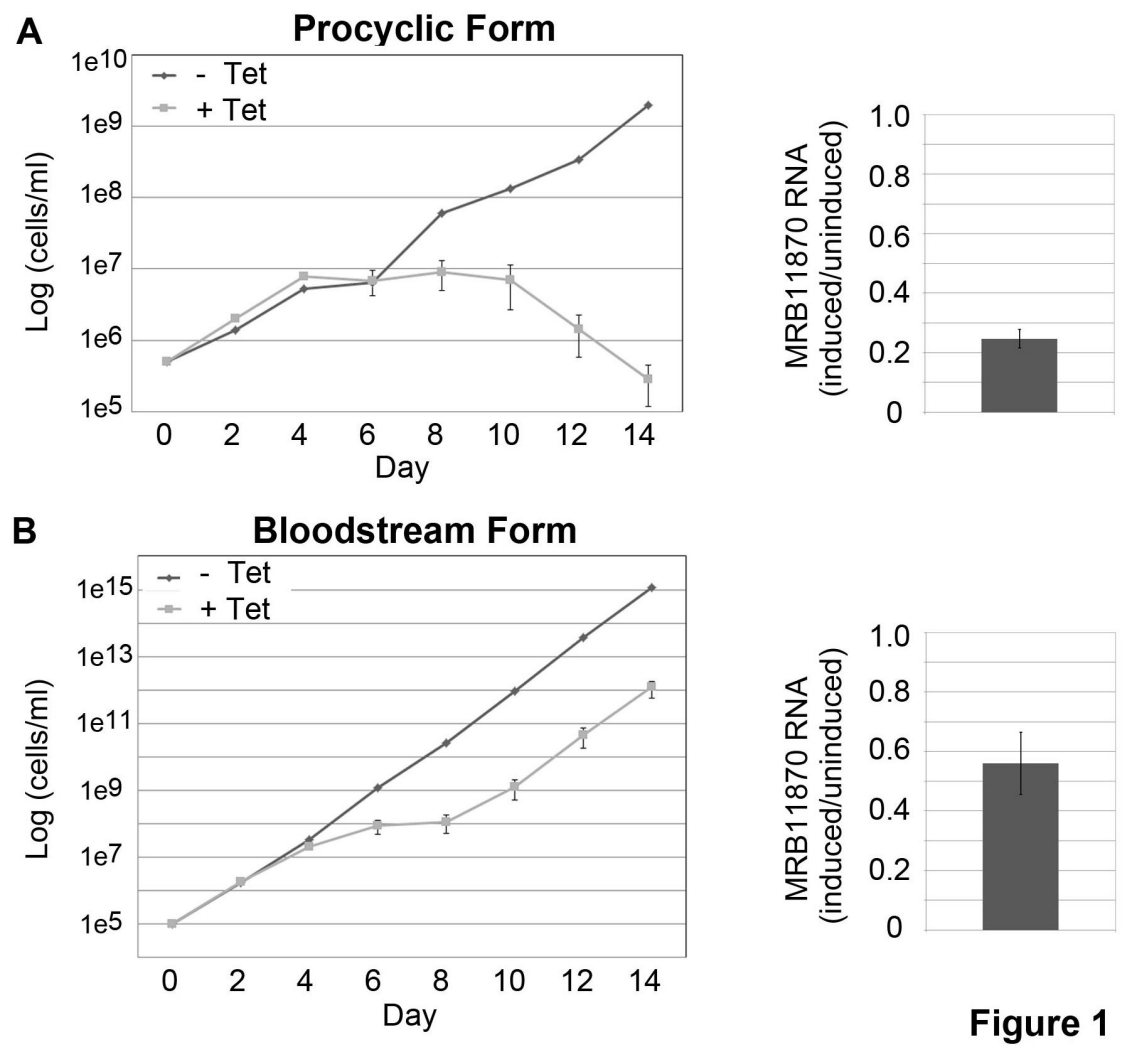
MRB11870 is essential for proliferation of both procyclic and bloodstream form *T. brucei*. MRB11870 was repressed by tetracycline-regulated RNAi in procyclic (A) and bloodstream (B) form *T. brucei*, and cell growth was monitored in triplicate cultures of uninduced and induced cells for 14 days. MRB11870 knockdown on day 4 post-induction was verified by qRT-PCR (n = 6) normalized to tubulin RNA.

 MRB11870 is a component of the MRB1 complex core, and other members of this complex are indispensible for growth and RNA editing in PF and BF *T. brucei* [10,13,16-19,21,24]. To determine whether MRB11870 is likewise essential for PF and/or BF proliferation, we silenced MRB11870 expression by conditional tetracycline-regulated RNAi in both life cycle stages and monitored cell growth for 14 days. Two clonal PF lines were isolated, and these behaved similarly. A representative growth curve is shown in Figure 1A. On day 4 following tetracycline addition to the medium, MRB11870 transcript levels were reduced to approximately 25% of wild type levels (Figure 1A, right). Uninduced and RNAi-induced cells grew similarly through day 6, at which time the MRB11870-repressed cells ceased growth. Starting on day 10 post-induction the MRB11870-repressed cells rapidly died. This is one of the most severe growth phenotypes reported for a protein involved in RNA editing to date. In the BF clone, MRB11870 mRNA was reduced to about 50% of wild type levels by day 4 upon RNAi-induction (Figure 1B, right). Proliferation slowed dramatically between days 4 and 8 post-induction, after which the cells appeared to escape the RNAi (Figure 1B, left), which is a typical phenomenon in this life stage [16,19]. Thus, MRB11870 is essential for proliferation of two *T. brucei* life cycle stages.

### MRB11870 is required for RNA editing

**Figure 2:**
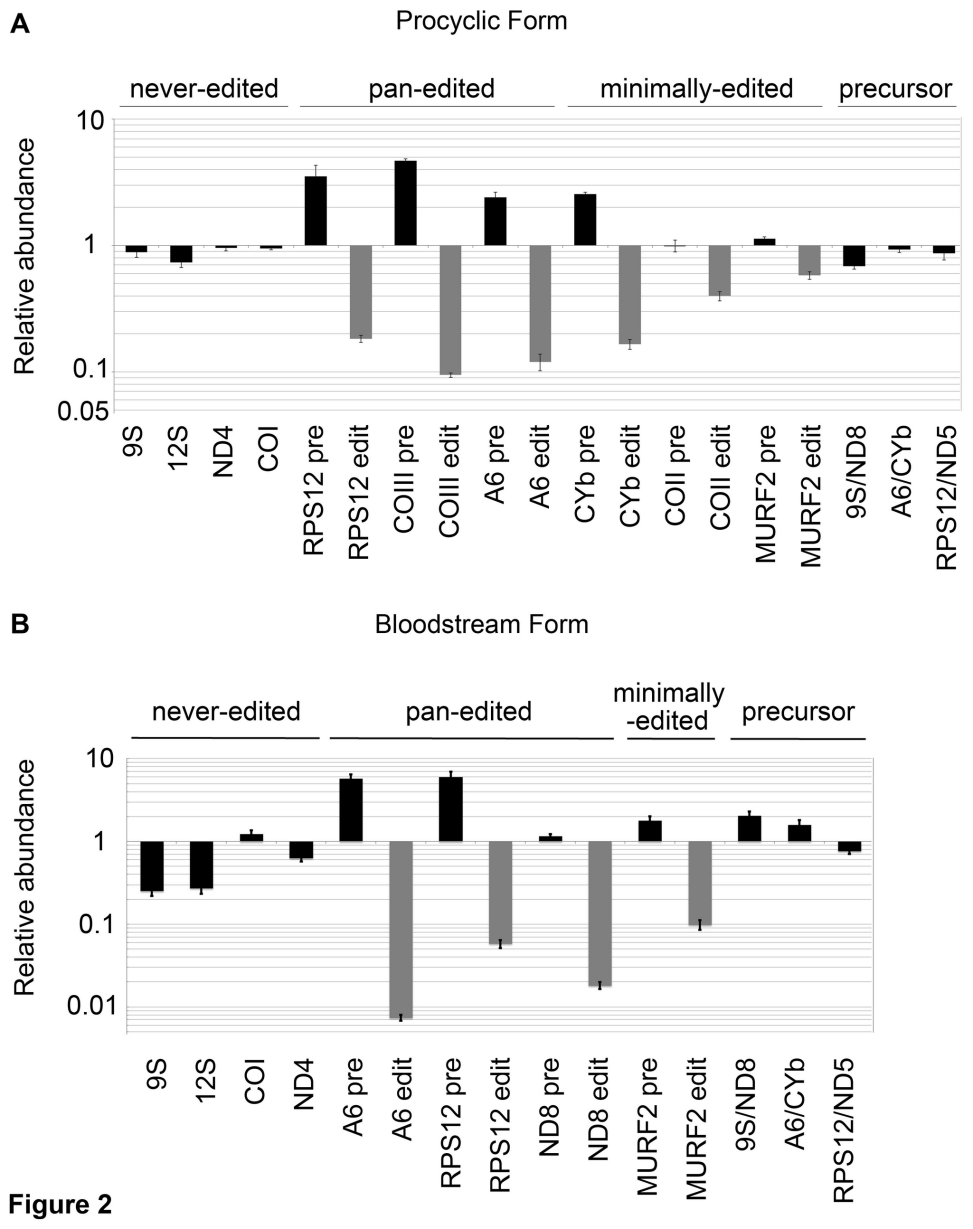
MRB11870 is required for editing of both pan-edited and minimally-edited mRNAs. RNA was isolated from procyclic (A) and bloodstream (B) form *T. brucei* on day 4 post-induction. RNAs were quantified by qRT-PCR using primer sets specific for selected never-edited, pan-edited, minimally-edited and dicistronic precursor RNAs. Relative RNA abundance indicates RNA levels in tetracycline-induced cells compared to those in uninduced cells. RNA levels were standardized to tubulin RNA and numbers represent the mean and standard error of 6-15 determinations.

 We next asked whether the essential phenotype of MRB11870 reflects a function for this protein in RNA editing. To examine the effect of MRB11870 repression on RNA editing, we isolated RNA from PF and BF trypanosomes in which the protein was either normally expressed or repressed via RNAi. We then measured the levels of numerous mitochondrial RNAs by quantitative (q) RT-PCR. We used a panel of primer sets that distinguish edited from pre-edited versions of a given mRNA [29]. We also measured RNAs that do not undergo editing, the so-called never-edited RNAs, and dicistronic pre-processed RNAs, to ask whether MRB11870 effects were specific to editing or extended to other aspects of RNA processing or stability [18]. Because editing of some RNAs is developmentally regulated, we examined a slightly different panel of RNAs in the two life cycle stages. Figure 2A shows the results from PF cells. This analysis demonstrated that the levels of never-edited 9S and 12S rRNAs, as well as never-edited ND4 and COI mRNAs, were unaffected by MRB11870 repression. Likewise, the levels of three dicistronic precursor transcripts spanning the 9S/ND8, A6/CYb and RPS12/ND5 genes remained unaltered. In contrast, all six edited mRNAs tested were dramatically impacted by repression of the MRB11870 protein. Edited versions of the pan-edited RPS12, COIII, and A6 mRNAs were decreased to between 10-20% of wild type levels. At the same time, the pre-edited versions of these same mRNAs were increased 2.2 to 4.5-fold. We also analyzed three mRNAs that are edited only within a small region, termed minimally-edited. This is of interest because some MRB1 complex proteins, such as TbRGG2, impact only pan-edited mRNAs, while core components reportedly affect the majority of edited mRNAs [13,16-19]. MRB11870 repression leads to a decrease in the levels of edited CYb, COII, and MURF2 mRNAs and a corresponding increase in pre-edited CYb mRNA. Thus, MRB11870 is required for editing of both pan-edited and minimally-edited mRNAs in PF trypanosomes, and appears to have little if any impact on RNA stability or processing.

 In BF trypanosomes, MRB11870 repression had a similar impact on RNA editing as that observed in PF. Editing of pan-edited A6, RPS12, and ND8 mRNAs and minimally-edited MURF2 mRNA was essentially abolished upon MRB11870 depletion. The corresponding pre-edited mRNAs accumulated to 5 to 6-fold their normal levels in the cases of A6 and RPS12 mRNAs, while ND8 and MURF2 pre-edited mRNAs exhibited minimal increases. We also observed that the abundance of three dicistronic precursors spanning the 9S/ND8, A6/CYb and RPS12/ND5 genes and the never-edited COI and ND4 mRNAs were largely unaffected by MRB11870 repression, again indicating the MRB11870 does not play any role in the processing or stabilization of these RNAs in this life cycle stage. However, we did observe an approximately 75% decrease in the levels of both 9S and 12S rRNAs upon MRB11870 repression in BF, although it is unclear whether this is a primary effect of MRB11870 as the same phenomenon was not observed in PF. Collectively, these data indicate that MRB11870 plays a broad and essential role in RNA editing in two stages of the *T. brucei* life cycle.

### MRB11870 impacts editing initiation, but does not dramatically affect gRNA levels

**Figure 3:**
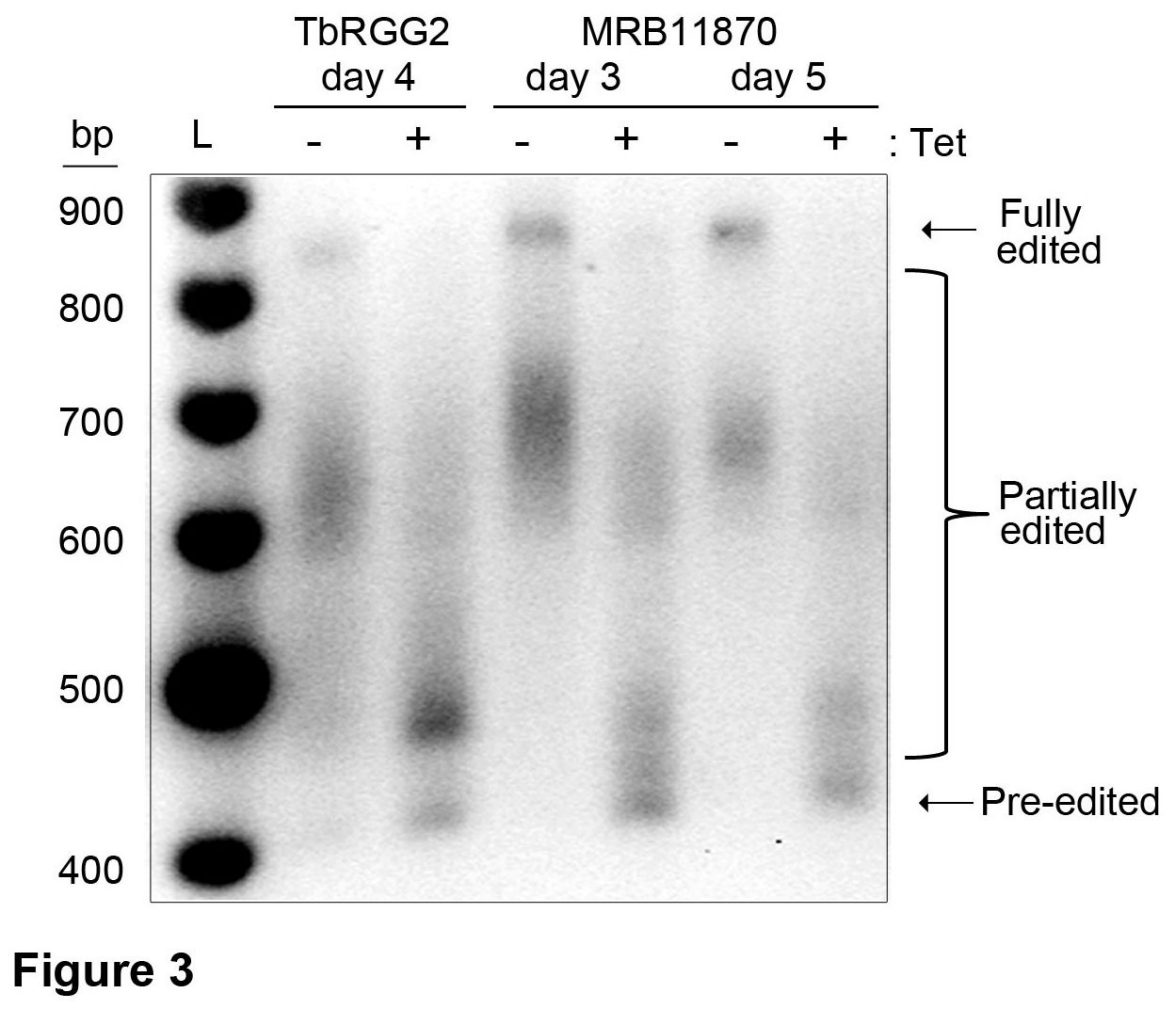
MRB11870 impacts an early step of the editing process. Agarose gel analysis of RT-PCR reactions using RNAs isolated from TbRGG2 and MRB11870 RNAi cells that were grown in the absence or presence of tetracycline (Tet) for the indicated number of days. Primers specific to the never-edited 5’ and 3’ ends of A6 mRNA, which flank the edited region, amplify the entire population of transcripts, including pre-edited, partially edited, and fully edited. L, size ladder.

Having shown that MRB11870 is essential for editing of both minimally-edited and pan-edited mRNAs, we next addressed which point of the process is affected when this protein is repressed. The qRT-PCR analysis of edited mRNAs described above monitors editing in regions near the 5’ ends of these RNAs [23,29]. Because editing takes place in a 3’ to 5’ direction, this analysis does not distinguish between impacts of protein repression at the initiation vs. progression stages of editing. Our observation that pre-edited mRNAs increase concomitantly for most transcripts (Figure 2) suggests some effect at the initiation of editing [17]. To further assess the effect of MRB11870 down-regulation at both the initiation and progression phases of the editing process, we analyzed the complete population of A6 mRNA from PF MRB11870 RNAi cells by RT-PCR using primers specific to the small 5’ and 3’ never-edited regions of the RNA, which flank the pan-edited region. This assay permits us to compare the relative amounts of pre-edited, partially edited, and fully edited mRNAs in uninduced and induced MRB11870 RNAi cells on the same gel [23,30]. We used this assay to compare the effect of MRB11870 repression to that of TbRGG2, which leads to a small decrease in editing initiation and a dramatic effect on the 3’ to 5’ progression of editing that manifests as accumulation of specific partially edited mRNAs even 6 days following induction of RNAi [17,23] (Figure 3). Compared to TbRGG2-repressed cells, flagellates ablated for MRB11870 exhibit a greater increase in pre-edited mRNA and lower accumulation of partially edited mRNA. This effect was evident by day 3 post-induction, and by day 5 pre-edited mRNA was the dominant species. Thus, because intermediate products do not build up, our results suggest that MRB11870 repression affects RNA editing initiation, perhaps due to an impact on utilization of the first gRNA. This phenotype is reminiscent of that observed upon MRB3010 depletion [17].

**Figure 4:**
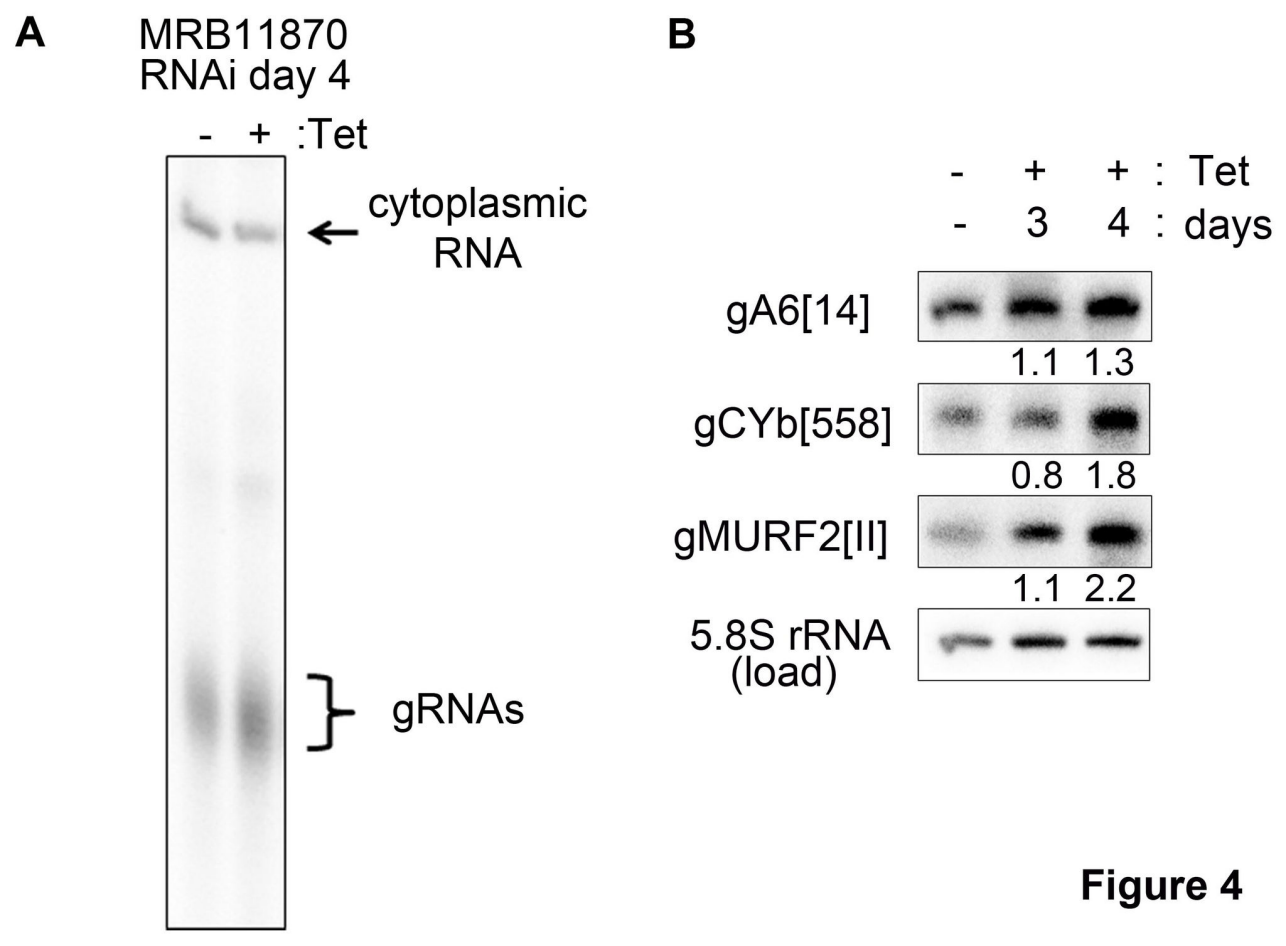
MRB11870 repression does not lead to a decrease in gRNA levels. (A) RNA was isolated from MRB11870 RNAi cells either uninduced (-) or induced with tetracycline (+) for 4 days, and labeled with alpha-[32P]-GTP and guanylyltransferase. The labeled gRNA population is indicated, as is a labeled cytoplasmic RNA used for normalization. (B) Six µg of RNA isolated from MRB11870 RNAi cells either uninduced (-) or induced with tetracycline (+) for 3 or 4 days was electrophoresed on 10% acrylamide/8 M urea gels and transferred to a nylon membrane. Membranes were probed with 5’-end labeled oligonucleotides complementary to gA6[14], CYb[558] or gMURF2[II] gRNAs plus 5.8S rRNA as a loading control. Signals were quantified by phosphorimaging and gRNA levels were normalized to the levels of 5.8S RNA from the same sample. The change in normalized gRNA levels at days 3 and 4 after RNAi induction are indicated below the blots.

Another MRB1 core component, the GAP1/2 heterotetramer, also affects RNA editing at the initiation stage, but this is a secondary effect of global gRNA destabilization upon GAP1/2 repression [13,16]. In contrast, repression of the MRB3010 core protein blocks editing initiation although gRNA levels remain normal [17]. To address whether the effect of MRB11870 on editing initiation reflects a decrease in gRNA abundance, we examined the levels of both the global gRNA population and specific gRNAs in the respective RNAi cells. In the experiment shown in Figure 4A, we isolated RNA from uninduced cells and cells that had been tetracycline-induced for 4 days. The total gRNA population was labeled by incubation of the RNA with alpha-[^32^P]-GTP and guanylyltransferase; a labeled cytoplasmic RNA serves as a loading control. These data clearly show that total gRNA levels are not decreased upon MRB11870 repression, and in fact gRNA abundance may be slightly increased. To further examine the impact of MRB11870 knockdown on gRNAs, we analyzed three specific gRNAs by Northern blot on days 3 and 4 after induction of RNAi, and normalized RNA abundance determined by densitometry to that of 5.8S rRNA (Figure 4B). Following quantification and normalization of specific gRNA levels, it was clear that gA6[14], gCYb[558], and gMURF2[II] levels were essentially unchanged on day 3 post-induction. The levels of these gRNAs increased on day 4 post-induction, with levels compared to uninduced cells ranging from 1.3 to 2.2-fold depending on the gRNA. From these data, we conclude that MRB11870 facilitates an early step in the editing process in a manner that is independent of gRNA stabilization.

### MRB11870 is essential for MRB1 complex protein-protein interactions

**Figure 5:**
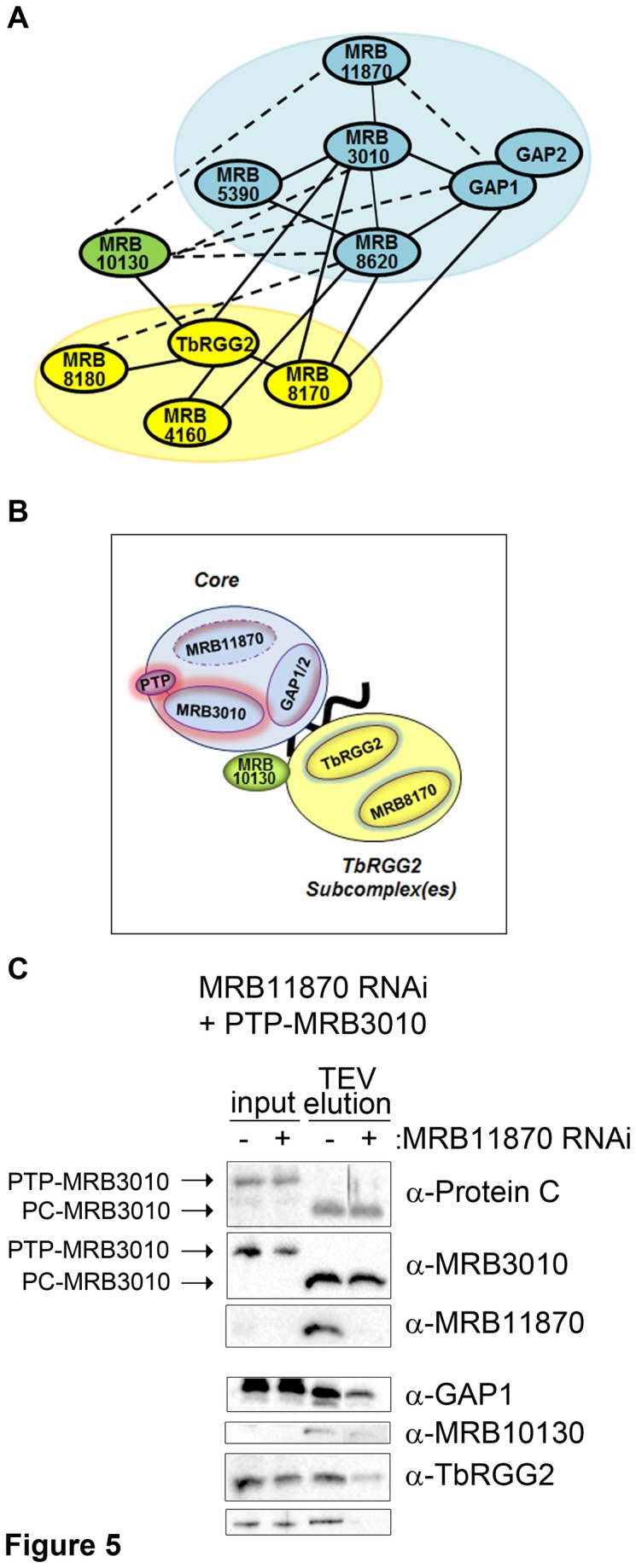
MRB11870 is required for numerous MRB1 complex macromolecular interactions. (A) Schematic showing the known strong (solid line) and weak (dotted line) yeast two-hybrid interactions between MRB1 components examined here (14). MRB1 core is indicated by the blue oval and TbRGG2 subcomplexes by the yellow oval. We omitted the strong two-hybrid interaction between MRB4160 and MRB8170 as these two proteins were later shown to engage in mutually exclusive interactions with TbRGG2 (21). (B) Cartoon of selected MRB1 complex components in cells harboring both MRB11870 RNAi (dotted line) and MRB3010 PTP-tagged at an endogenous allele (pink highlight). Shown in the figure is the MRB1 core (blue), from which GAP1/2 may transiently dissociate. Blank oval indicates additional core proteins including MRB5390 and MRB8620. Also depicted are the TbRGG2 subcomplex(es) (yellow), a subset of which contain MRB8170 as depicted. The MRB10130 protein, which may act as an MRB1 complex organizer is shown in green. Black line indicates RNA, which may include gRNA, mRNA or both. (C) PTP-MRB3010 and associated proteins were isolated by IgG Sepharose chromatography and TEV protease cleavage from RNase-treated extracts of cells either uninduced (-) or tetracycline-induced (+) for MRB11870 RNAi. PTP-MRB3010 and PC-MRB3010 indicate the tagged protein before and after TEV cleavage, respectively. Both input and TEV elutions were analyzed by western blot for MRB1 complex components using the antibodies indicated on the right.

We previously defined the architecture of the MRB1 complex, which includes a core complex of at least 6 proteins, including GAP1/2; the latter may also exist as a separate heterotetramer and recruit gRNAs to the core [14] (Figure 5A). Yeast two-hybrid data indicate that MRB11870 interacts within the core primarily through a strong direct interaction with MRB3010, with a possible contribution from a weak direct interaction with GAP1. Additional components of the MRB1 complex include the TbRGG2 subcomplex(es), which contain the eponymous protein and a still imprecisely characterized combination of the MRB8170, MRB4160 and/or MRB8180 proteins [14,21]. MRB10130 is an Armadillo repeat protein that interacts with a large number of MRB1 proteins, including components of both the core and the TbRGG2 subcomplex(es), and may serve as an MRB1 organizer. Towards understanding the function of MRB11870 in RNA editing, we next wanted to address its macromolecular interactions within the MRB1 complex. To this end, we generated PF cells harboring both an endogenously PTP-tagged allele of the MRB3010 core component and the MRB11870 RNAi construct (Figure 5B). This set up allowed us to perform pulldowns of the MRB1 core in the presence and absence of MRB11870 and determine whether the depletion of the latter protein dramatically impacts the composition of the core and/or its interactions with other MRB1 subcomplexes and proteins.

PTP-MRB3010 and associated proteins were isolated from RNase-treated MRB11870-expressing or repressed cell extracts by affinity chromatography on IgG Sepharose, which binds the Protein A moiety of the PTP tag, followed by a subsequent TEV protease cleavage (Figure 5C). The resulting Protein C (PC)-tagged MRB3010 was detected in the eluates by both anti-Protein C and anti-MRB3010 antibodies, and MRB11870 knockdown was verified using anti-MRB11870 antibodies. Eluates from uninduced and MRB11870-ablated cells were normalized to PC-MRB3010 levels, and the abundance of selected MRB1 complex components was analyzed by western blot. Since GAP1/2 is a critical, gRNA binding component of the core that weakly interacts with MRB11870, we first asked whether association with the MRB1 core of GAP1, serving as a proxy for both GAP proteins, was affected by MRB11870 repression. Figure 5C shows that the amount of GAP1 associated with the MRB1 core was significantly decreased when MRB11870 was repressed, although the steady state level of the protein remains unaffected (input). MRB11870 may be directly involved in the association of GAP1/2 with the core, as suggested by the yeast two-hybrid data showing a weak direct MRB11870-GAP1 interaction [14]. Additionally, the absence of MRB11870 may cause a general destabilization of the MRB1 core, or it may perturb the structures of MRB3010 and/or MRB8620, core proteins that display strong direct interactions with GAP1. We next asked whether association of the MRB1 core with MRB10130, the putative organizer protein, is impacted by MRB11870 repression. As shown in Figure 5C, core-associated levels of MRB10130 are also substantially decreased upon MRB11870 repression, possibly reflecting the direct interaction between these two proteins and/or global disruption of core structure. 

Finally, we asked whether association of TbRGG2 subcomplexes with the core was impacted by MRB11870 repression. Western blot analysis of TbRGG2 and its binding partner MRB8170 revealed a strong disruption of the core-TbRGG2 subcomplex association in the absence of MRB11870. Interestingly, MRB11870 does not appear to engage in direct interactions with either TbRGG2 or MRB8170 [14]. Thus, the dramatic decrease in the association of these proteins with the core may be a secondary consequence of MRB10130 reduction, since this putative organizer directly contacts both the core and TbRGG2 (Figure 5A) [14]. Additionally, loss of GAP1/2 and its associated gRNAs may lead to decreased interaction with TbRGG2-bound mRNA that promotes the association of core and TbRGG2 subcomplexes [20]. Together, the protein-protein interaction studies reveal a pivotal role for MRB11870 in the MRB1 complex structure and highlight the intricate nature of the macromolecular interactions that comprise the MRB1 complex.

## Discussion

 MRB1 is a dynamic multiprotein complex in trypanosome mitochondria, numerous components of which are critical for the vital and unique RNA editing process [10]. Our previous studies, employing yeast two-hybrid and *in vivo* pulldown analyses, defined an MRB1 complex core of at least six proteins that interact in an RNA independent manner [14]. In this manuscript, we characterize MRB11870, a protein component of the MRB1 core, and demonstrate that it is essential for growth and RNA editing in two *T. brucei* life cycle stages. MRB11870 affects the initiation of the editing process, despite the presence of abundant gRNAs. Comparative *in vivo* pulldown studies in cells with normal or repressed MRB11870 levels also reveal a key structural role for MRB11870 within the MRB1 complex. 

 Proteins comprising the MRB1 complex core that have been characterized to date have two distinct phenotypes. The GAP1/2 heterotetramer binds and stabilizes gRNAs [13,15,16]. Repression of either GAP1 or GAP2 destabilizes both proteins, leading to a dramatic decrease in gRNA levels and a consequent halt in RNA editing [13,16]. On the other hand, repression of MRB3010 does not affect gRNA levels, but nevertheless leads to a significant inhibition of RNA editing of both pan- and minimally-edited mRNAs [17]. The phenotype of cells repressed for MRB11870 reported here is similar to that of MRB3010 knockdowns, with a broad and significant effect on editing initiation but no decrease in gRNA levels. This observation is consistent with our reported protein-protein interaction studies, in which the only MRB1 component exhibiting a strong, direct interaction with MRB11870 was MRB3010 [14]. Thus, the functional analysis of MRB11870 reported here validates aspects of our previously published model of MRB1 architecture derived from comprehensive yeast two-hybrid and *in vivo* pulldown studies. Together, the current data support a model in which GAP1/2 are the gRNA binding component of the core, while additional core components, including MRB11870 and MRB3010, are required for the functional association of gRNA with the editing machinery. Interestingly, gRNA levels are modestly increased by day 4 following MRB11870 RNAi induction, representing a hitherto unseen phenotype. This increase in gRNAs may be a consequence of their decreased utilization in the editing process when MRB11870 is repressed, suggesting that gRNAs are consumed during editing and accumulate if editing is abrogated. The mechanisms governing the fate of gRNAs used in editing are not understood. The gRNA cycle and the precise roles of MRB1 complex components in gRNA utilization, and possibly destruction, will be important avenues for future research.

 Downregulation of MRB11870 dramatically affected macromolecular interactions within the MRB1 complex. Western blot analysis of GAP1 revealed that, while its total levels in the organelle are unaffected by MRB11870 repression, the fraction of GAP1 associated with the MRB3010 core protein is significantly diminished when MRB11870 is repressed. MRB11870 may be directly involved in maintaining GAP1/2 association with the core, through a weak direct interaction between the GAP1 and MRB11870 [14]. Alternatively or in addition, the inability of GAP1 to properly associate with the core in the absence of MRB11870 may be indicative of an overall disturbance of the core structure. These data also demonstrate that the ability of GAP1/2 to stabilize the total gRNA population is not dependent on the proteins’ interaction with the rest of the MRB1 core, since gRNAs remain abundant when the GAP1/2 association with MRB3010 is impaired in MRB11870-repressed cells. The decreased interaction of Armadillo repeat protein MRB10130 with the core upon MRB11870 ablation may also reflect the reported weak direct interaction between these two proteins and/or altered core structure. While neither TbRGG2 nor MRB8170 interact directly with MRB11870, both make direct contact with other core proteins, and so their decreased interaction with the core could result from global core disruption [14]. Moreover, MRB10130 directly interacts with both TbRGG2 and numerous core components and may serve as a bridge or promote their interaction (Figures 5A and B) [14]. Finally, decreased association of TbRGG2/MRB8170 with the core could result, at least in part, from disruption of gRNA/mRNA interactions responsible for the RNA-enhanced association of core with TbRGG2 subcomplexes [14]. Although the pulldown experiments reported here were performed in RNase-treated extracts, RNA may still facilitate interaction between the MRB1 core and TbRGG2 subcomplex *in vivo*, but be dispensable once this interaction is established. Both TbRGG2 and MRB8170 exhibit RNA binding activity *in vitro*, with significantly stronger affinities for mRNA than for gRNA, suggesting that TbRGG2/MRB8170 containing subcomplexes may play a role in mRNA utilization during editing, including a role in gRNA/mRNA annealing [20,21]. Thus, loss of GAP1-bound gRNA from the core upon MRB11870 repression could impact association of mRNAs bound to TbRGG2 and/or MRB8170. Regardless of the precise mechanisms, the dramatic disruption of macromolecular associations within the MRB1 complex when MRB11870 is repressed highlights the many coordinated interactions comprising this dynamic complex.

 The biochemical function of MRB11870 is not known, but its characterization as a member of the pentein superfamily may yield some clues in this regard. The non-catalytic eIF6, which functions in both ribosome assembly and translation [28,31], represents the simplest form of a pentein domain-containing protein. In the nucleus, eIF6 associates with pre-60S ribosomal subunits and is necessary for biogenesis of mature 60S subunits, including the proper processing of pre-rRNA precursors [32]. An analogous role for MRB11870 in MRB1 core assembly would be consistent with the broken MRB3010-GAP1 interaction that we demonstrate in MRB11870 repressed cells. The second function of eIF6 is to act as a ribosome anti-association factor, sterically blocking the interaction between 60S and 40S ribosomal subunits at critical points in the ribosome cycle. During protein synthesis, GTP hydrolysis catalyzed by 60S-bound ELF1 causes a conformational change in the 60S subunit, promoting eIF6 release and subunit joining [33]. One could envision a similar role for MRB11870 in regulating interactions between MRB1 subcomplexes. Catalytic pentein proteins are larger than non-catalytic members of the family due to the appendage of numerous loops and extensions, and they range from approximately 400-660 amino acids [27]. Interestingly, MRB11870 is significantly larger (310 amino acids) than the minimal pentein protein eIF6 (108 amino acids), suggesting it might have a catalytic function. Catalytic pentein proteins include diverse guanidine-modifying enzymes, such as hydrolases, amidinotransferases, and dihydrolases. Peptidylarginine demininases (PADs) are one member of this family. PADs convert arginine and monomethylarginine (MMA) residues in proteins to citrullines, with a subsequent dramatic effect on protein function [34]. We have identified methylarginine on several MRB1 complex proteins, including TbRGG2, MRB10130, and MRB4160 [35]. While the functions of these modifications are currently unknown, both citrullination of the arginine residues, which would preclude their methylation, as well as citrullination of MMA would likely modulate macromolecular interactions. Investigating the potential enzymatic activities of MRB11870 and determining its precise role in modulating the dynamic interactions that comprise the MRB1 complex will be exciting topics for future research. 

## Materials and Methods

### Generation of cell lines and growth curves

 To generate MRB11870 RNAi cells, the entire open reading frame of MRB11870 was amplified with forward primer CAGGATCCATGCTGCGCCACACATCACG and reverse primer CAAAGCTTTTTCTGCAGTTGATGCGTCTGC and cloned into the BamHI and HindIII restriction sites (underlined) of the p2T7-177 plasmid [36] between opposing tetracycline-regulated T7 RNA polymerase promoters. The resulting construct was linearized with NotI and transfected into parental procyclic strain 29-13 and bloodstream form single marker strain *T. brucei* [37]. Transformants were selected with 2.5 µg/ml phleomycin and clones were obtained by limiting dilution. To generate the cell line harboring constructs for both MRB11870 RNAi and PTP-tagged MRB3010, a previously described PTP-MRB3010 plasmid [17] was linearized with BpiI and electroporated into the clonal MRB11870 RNAi line. Cells were selected with 2.5 µg/ml phleomycin and 1 μg/ml puromycin and cloned by limiting dilution. Repression of MRB11870 was induced in all cell lines by adding 2.5 µg/ml tetracycline to cell cultures. TbRGG2 RNAi cells were previously described [19].

### Quantitative real time PCR

 RNA was isolated from uninduced and tetracycline-induced PF and BF MRB11870 RNAi cells on day 4 post-induction using Trizol, and qRT–PCR reactions were performed with primers specific to mitochondrial transcripts as previously described [19,29]. The level of MRB11870 mRNA depletion was analyzed by qRT-PCR (n = 6) with primers MRB11870 qRT-UTR Rev 5’ CGATCTGATACGTTCGTGGTTTGC 3’ and MRB11870 qRT-UTR Fwd 5’ GTGTGCTCATGGTTGTGGTTAG 3’. These primers target the nts 42-135 following the stop codon within the reported 3’ UTR [38,39]. Reactions were normalized to the level of tubulin cDNA.

### RT-PCR analysis of A6 transcripts

 RNA was isolated from uninduced and tetracycline-induced PF TbRGG2 RNAi cells and MRB11870 RNAi cells and DNase-treated as described previously [23]. Oligo-dT primed cDNA was prepared by reverse transcription with Superscript III (Invitrogen) by the manufacturer’s instructions. A6-specific primers A6 5’ NE (5’ GCGAATTCAAATAAGTATTTTGATATTATTAAAG 3’) and A6 3' (5' ATTAACTTATTTGATCTTATTCTATAACTCC 3’) that anneal to the never edited 5’ and 3’ regions that flank the edited part of the A6 mRNA were used for RT-PCR analysis (restriction site underlined).

### Guanyltransferase labeling and northern blot analyses of RNA

Quantification of the total gRNA population by guanylyltransferase labeling was performed as previously described [17]. For northern blot analysis, total RNA (6 µg) was dissolved in loading dye (90% formamide, 10 mM EDTA, 0.05%xylene cyanol, 0.05% bromophenol blue), heated for 5 min to 65°C and resolved by 8 M urea/10% PAGE. RNA was transferred to positively charged nylon membrane (Boehringer Mannheim) by electrotransfer in 0.5X TBE and UV crosslinked using a Stratalinkner (Stratagene). DNA oligonucleotide probes (10 pmol) were labeled with ϒ-[^32^P] ATP (6000 Ci/mmol) using T4 kinase. Membranes were prehybridized in ULTRAhyb-oligo solution (Ambion) for 30 minutes and then hybridized with the probe overnight at 42°C. The membrane was washed 3 times for 30 minutes each with 20 mL of 4xSSPE, 0.5%SDS at 42°C and was exposed to a phosphor screen overnight. The screen was scanned with the Bio-Rad Personal Imager FX and the quantitation of the density of bands in northern blots was performed with the Quantity One software (Bio-Rad). The following 5’- ^32^P-labeled oligonucleotides were used:

gA6[14]: ATAATTATCATATCACTGTCAAAATCTGATTCGTTATCGGAGTTATAGTATAT

gMURF2[II] gRNA (maxicircle encoded): CATTCAATTACTCTAATTTAATTTTATTTTTGTGC

gCYb[558]: GCGGATCCTTATTCCCTTTATCACC

Pre-5.8S rRNA: CCATCGCGACACGTTGTGGGAGCCG

### Immunoprecipitation and western blot analysis

 PF cells expressing both the endogenous PTP-MRB3010 and the tetracycline-inducible MRB11870 RNAi constructs were either grown in the presence or absence of the tetracycline for 4 days. Expression of PTP-MRB3010 was verified by western blot using affinity purified rabbit anti-protein C (ICL). MRB11870 knockdown was verified by qRT-PCR at the RNA level as described above and by western blot with anti-MRB11870 antibodies (which are effective only in partially purified samples but not in whole cell extracts). Whole cell extracts were nuclease treated and MRB3010 and its associated proteins were affinity purified by IgG Sepharose 6 Fast Flow chromatography and TEV protease cleavage as described previously [14]. Input and TEV cleavage samples were subsequently analyzed by western blot. Whole cell extracts and TEV elutions were electrophoresed on 10% SDS-polyacrylamide gels and proteins were transferred onto nitrocellulose membrane. Membranes were probed with previously described polyclonal antibodies against MRB3010 [14], MRB11870 [14], MRB8170 [14], GAP1 [17] and TbRGG2 [19]. Polyclonal antibodies against MRB10130 were produced by Bethyl laboratories against the oligopeptide CDLQVLLQRGAVEPGAAPGVM. Western blots were analyzed by chemiluminescence using a Chemidoc MP System (Bio-Rad). Normalization of PC-MRB3010 protein amounts using anti-Protein C antibody was performed with Image lab Software. This experiment was performed twice, using biological replicates, with similar results.
